# Novel recombinant avian infectious bronchitis viruses from chickens in Korea, 2019–2021

**DOI:** 10.3389/fvets.2023.1107059

**Published:** 2023-02-01

**Authors:** Hyun-Jin Kim, Hyuk-Chae Lee, Andrew Y. Cho, Yun-Jeong Choi, Heesu Lee, Dong-Hun Lee, Chang-Seon Song

**Affiliations:** ^1^Avian Diseases Laboratory, College of Veterinary Medicine, Konkuk University, Seoul, South Korea; ^2^KHAV Co., Ltd., Seoul, South Korea; ^3^Wildlife Health Laboratory, College of Veterinary Medicine, Konkuk University, Seoul, South Korea

**Keywords:** infectious bronchitis virus, recombination, pathogenicity, antigenicity, tissue tropism

## Abstract

Infectious bronchitis virus (IBV) has evolved through various mutation mechanisms, including antigenic drift and recombination. Four genotypic lineages of IBVs including GI-15, GI-16, GI-19, and GVI-1 have been reported in Korea. In this study, we isolated two IBVs from chicken farms, designated IBV/Korea/289/2019 (K289/19) and IBV/Korea/163/2021 (K163/21), which are two distinct natural recombinant viruses most likely produced by genetic reassortment between the S1 gene of K40/09 strain (GI-19 lineage) and IBV/Korea/48/2020 (GI-15 lineage) in co-infected commercial chickens. Comparative sequence analysis of hypervariable regions (HVRs) revealed that the K289/19 virus had similar HVR I and II with the K40/09 virus (100% and 99.2% nucleotide sequence identity, respectively), and HVR III with the IBV/Korea/48/2020 virus (100% nucleotide sequence identity). In contrast, the K163/21 virus had HVR I and II similar to the IBV/Korea/48/2020 virus (99.1% and 99.3% nucleotide sequence identity, respectively), and HVR III to the K40/09 virus (96.6% nucleotide sequence identity). The K289/19 virus exhibited similar histopathologic lesions, tissue tropism in trachea and kidney, and antigenicity with the parental K40/09 virus. The K163/21 exhibited similar pathogenicity and tissue tropism with the K40/09 virus, which were similar results with the isolate K289/19. However, it showed a lower antigenic relatedness with both parental strains, exhibiting R-value of 25 and 42, respectively. The continued emergence of the novel reassortant IBVs suggests that multiple recombination events have occurred between different genotypes within Korea. These results suggest that antigenic profiles could be altered through natural recombination in the field, complicating the antigenic match of vaccine strains to field strains. Enhanced surveillance and research into the characteristics of newly emerging IBVs should be carried out to establish effective countermeasures.

## 1. Introduction

Infectious bronchitis virus (IBV) is a pathogen associated with acute respiratory tract diseases in chickens. The IBV is highly contagious and can also affect multiple organs according to their pathotypes, such as the respiratory tract, kidneys, and reproductive tract. Chickens infected with IBV show respiratory signs, reduced egg production, weight loss, and decreased weight gain. Mortality may vary depending on the IBV strain, secondary bacterial infection, or coinfection with other viruses. It is economically important to control this infection because of its detrimental effects on poultry production (1, 2).

High antigenic diversity of IBVs and the extensive emergence of variants are problematic, resulting in poor cross-protection by the available vaccine strains (3–5). The spike (S) protein of IBV, comprising ~3.4 kb, is a major inducer of virus-neutralizing antibodies and an important factor in determining tissue tropism (6), which is post-translationally cleaved to S1 and S2 subunits. The S2 subunit is associated with membrane fusion (6, 7). The S1 subunit, responsible for host cell attachment, has a receptor binding domain and hypervariable regions (HVR). HVRs (HVR I, II, and III) are associated with neutralizing antibodies, virulence of the virus, and tissue tropism (8–12). The HVR I is a major epitope inducing neutralizing antibodies and associated with tissue tropism for respiratory tract (12, 13). In addition, a previous study reported that the HVR II is related to kidney affinity (14). Because of these characteristics of the S1 subunit, IBV genotypes have been classified based on the S1 gene (15–17).

According to the classification system of IBV lineages defined by Valestro et al. (15), IBVs isolated in Korea are classified into four genotypic lineages: GI-15, GI-16, GI-19, and GVI-1 (16). The GI-15, previously designated as Korean group I (K-I), is associated with respiratory diseases in chickens (18). The GI-19 includes nephropathogenic viruses that can be divided into three subgroups: KM91-like, QX-like, and K40/09-like (17). The KM91-like subgroup, also known as Korean group II a (K-IIa), have been detected from 1990's with nephropathogenicity in chickens (19, 20). The QX-like subgroup, also known as Korean group II b (K-IIb), causes outbreaks in chickens globally, including South Korea (15, 17). The K40/09-like subgroup was reported as the Korean new cluster I in our previous study, which is produced by recombination between the KM91-like virus and QX-like virus (21). The GI-16 strains were isolated in Korea during 2003–2006, designated as Korean group III (K-III) (20), which have caused respiratory syndrome and nephropathogenic diseases in chicken farms (22, 23). The GVI-1, reported as the Korean new cluster II causes clinical signs mainly in the respiratory tract of chickens (24, 25). Diverse recombinant genotypes of IBVs in Korea suggests that multiple recombination have occurred between different genotypes in Korea.

In this study, we isolated two novel IBVs which are natural recombinant between the GI-15 and GI-19 lineages. We investigated their pathobiological characteristics, such as pathogenicity, antigenicity, and tissue tropism in chickens.

## 2. Materials and methods

### 2.1. Virus isolation and propagation

The IBVs used in this study were K40/09, IBV/Korea/48/2020, K289/19, and K163/21. The K40/09 (K40/09-like subgroup of GI-19) and IBV/Korea/48/2020 (B4-like subgroup of GI-15) viruses were isolated from chickens in Korea (17, 21). The isolate K289/19 was isolated from 46-day-old broiler breeders showing respiratory signs and nephritis. The isolate K163/21 was isolated from a 77-day-old layer that showed respiratory signs. All IBVs used in this study were propagated in 10-day-old specific-pathogen free (SPF) embryonated chicken eggs for 48 h (21). The allantoic fluid was harvested from the inoculated eggs and stored at −70°C until use. Before using the allantoic fluid, quantification of viruses was conducted by titration to calculate 50% embryo infectious dose (EID_50_) (26).

### 2.2. PCR, sequencing, and phylogenetic analysis

Viral RNA was extracted from harvested allantoic fluid using the RNeasy Mini Kit (Qiagen, Hilden, Germany) according to the manufacturer's instructions. The S1 gene of each IBV isolate was amplified using a OneStep RT-PCR Kit (Qiagen) according to the manufacturer's instructions and using two pairs of primers (S1 forward F: CGGAACAAAAGACMGACTTAGT and S1 forward R: CWGTACCATTAACAAARTAAGCMAG; S1 rear F: TGTGTATTTTAAAGCAGGTGGACC and S1 rear R: GTTTGTATGTACTCATCTGTAAC). The reaction was conducted in a ProFlex PCR System (Applied Biosystems, Forest City, CA, USA) at 50°C for 30 min; 95°C for 15 min, and 35 cycles of 94°C for 60 s, 53°C for 60 s, 72°C for 120 s, and a final extension step at 72°C for 7 min. The PCR products were purified using a GeneJET Gel Extraction Kit (Thermo Fisher Scientific, Waltham, MA, USA) and sequenced using Sanger sequencing (Macrogen Co., Ltd., Seoul, South Korea). The obtained nucleotide sequences of the S1 gene were assembled and aligned with the prototype of different lineages of IBV and reference strains (15, 17) shown in Supplementary Table S1 using Geneious Prime^®^ 2022.1.1 software (https://www.geneious.com/). A phylogenetic tree was constructed in MEGA version 10.2.5, using the neighbor-joining method with 1000 bootstrap replicates.

### 2.3. Recombination analysis

The Recombination Detection Program (RDP) 4 software (v. 4.39) was used to identify putative recombination events using several detection methods: RDP, GENECONV, BootScan, MaxChi, Chimera, SiScan, Phylpro, LARD, and 3Seq (27). Recombination events where at least five detection methods showed a *p*-value < 1 × 10^−14^ were accepted (28). After identifying their putative parental strains from 59 reference sequences, the S1 gene sequences of the IBV isolates were compared with the sequences of their putative parental strains. Nucleotide sequences from the S1 gene were used to generate similarity plot using the Simplot software (v. 3.5.1), with a window size of 200 bp and a step size of 20 bp (29).

### 2.4. Pathogenicity and tissue tropism investigation

One-day-old SPF chickens (*n* = 150) were randomly divided into five groups (Group 1: K40/09; Group 2: IBV/Korea/48/2020; Group 3: K289/19; Group 4: K163/21; Group 5: Negative control; n=30 in each group) and maintained separately in isolation cabinets (Three Shine, Daejeon, Korea). All chickens used in this study were obtained from Namduk SPF (Incheon, Republic of Korea). Chickens in the treated group were inoculated with a virus with a 10^5^ EID_50_ per chick *via* the ocular route. Chickens from the negative control group were inoculated with sterile phosphate-buffered saline (PBS) for the same volume as the virus inoculated groups. At 5 days post-inoculation (5 dpi), twenty chicks were sacrificed from each group. The upper, middle, and lower regions of tracheal and kidney tissues were collected from ten necropsied chickens from each group for histopathological examination. Ciliary loss, inflammatory response of the trachea, and severity of kidney inflammation were scored as previously described (30). Tracheal and kidney tissues were collected from remaining 10 sacrificed chickens from each group to isolate virus. Tracheal and kidney tissues were homogenized and diluted to 10% (w/v) in PBS containing 400 mg/mL gentamicin. The supernatants of the homogenized tissue samples were clarified by centrifugation at 3,000 rpm (107 × g) for 10 min, filtered using a 0.45 μm Minisart syringe filter (Sartorius, Göttingen, Germany), followed by propagationin 10-day-old SPF embryonated chicken eggs at 37°C for 72 h. After incubation, the allantoic fluid was harvested and viral RNA was extracted. Viral replication in trachea and kidney samples was confirmed using real-time reverse transcription-PCR (rRT-PCR) as previously described (31).

Ten of thirty chicks from each group were observed for clinical signs, morbidity, and mortality for 2 weeks after experimental infection. Clinical signs were monitored twice daily for clinical signs, including rales, nasal discharge, coughing, eye irritation, depression, and watery diarrhea. The morbidity and mortality rates were monitored daily. If mortality occurred, the dead chickens were necropsied to observe gross lesions.

### 2.5. Antigenicity study using cross-neutralization test

Allantoic fluids of the K40/09, IBV/Korea/48/2020, K289/19, and K163/21 viruses were inactivated with 0.1% formaldehyde for 24 h at room temperature (20–22°C). Inactivated allantoic fluids were emulsified with Montanid ISA70 (Seppic, Paris, France) at ratio of 3:7 (v/v) to be injected into SPF chickens to produce antisera (32).

Two-week-old SPF chickens (*n* = 20) were randomly separated into four groups and each oil-emulsified inactivated virus (0.5 mL) was intramuscularly inoculated into each group. Two weeks after inoculation, all groups were given a booster of the same inactivated virus. Two weeks after boosting, hyperimmune antisera were collected from each group and inactivated at 56°C for 30 min. Virus cross-neutralization tests were performed using SPF embryonated eggs with the Alpha method according to the OIE Terrestrial Manual (33). Briefly antisera and the ten-fold dilutions of the allantoic fluids, which began from the titer of 10^7^ EID_50_, were mixed and incubated at 37°C for 30 min. After incubation, the mixtures were inoculated into five SPF embryonated chicken eggs at each dilution. Eggs were inoculated with the corresponding titers of the virus with PBS in parallel. Endpoints were calculated using the Reed and Muench method (34). The neutralization index (NI) and antigenic relatedness (R-value) were calculated as previously described (19).

### 2.6. Statistical analyses

The ciliary loss and inflammatory response scores of test groups were compared with the score of negative group using Dunnett's multiple comparisons test. Re-isolation rate of the inoculated virus among the groups was analyzed using one-tailed Fisher's exact test. Statistical significance was set at *p* < 0.05. All statistical analyses were performed using GraphPad Prism version 9.2.0 software (San Diego, CA, USA).

### 2.7. Ethics statement

The animal study was reviewed and approved by the Institutional Animal Care and Use Committee (IACUC) of Konkuk University of South Korea (permission number KU22113, July 18, 2022).

## 3. Results

### 3.1. Phylogenetic, recombination and genomic analysis

**Figure 1:**
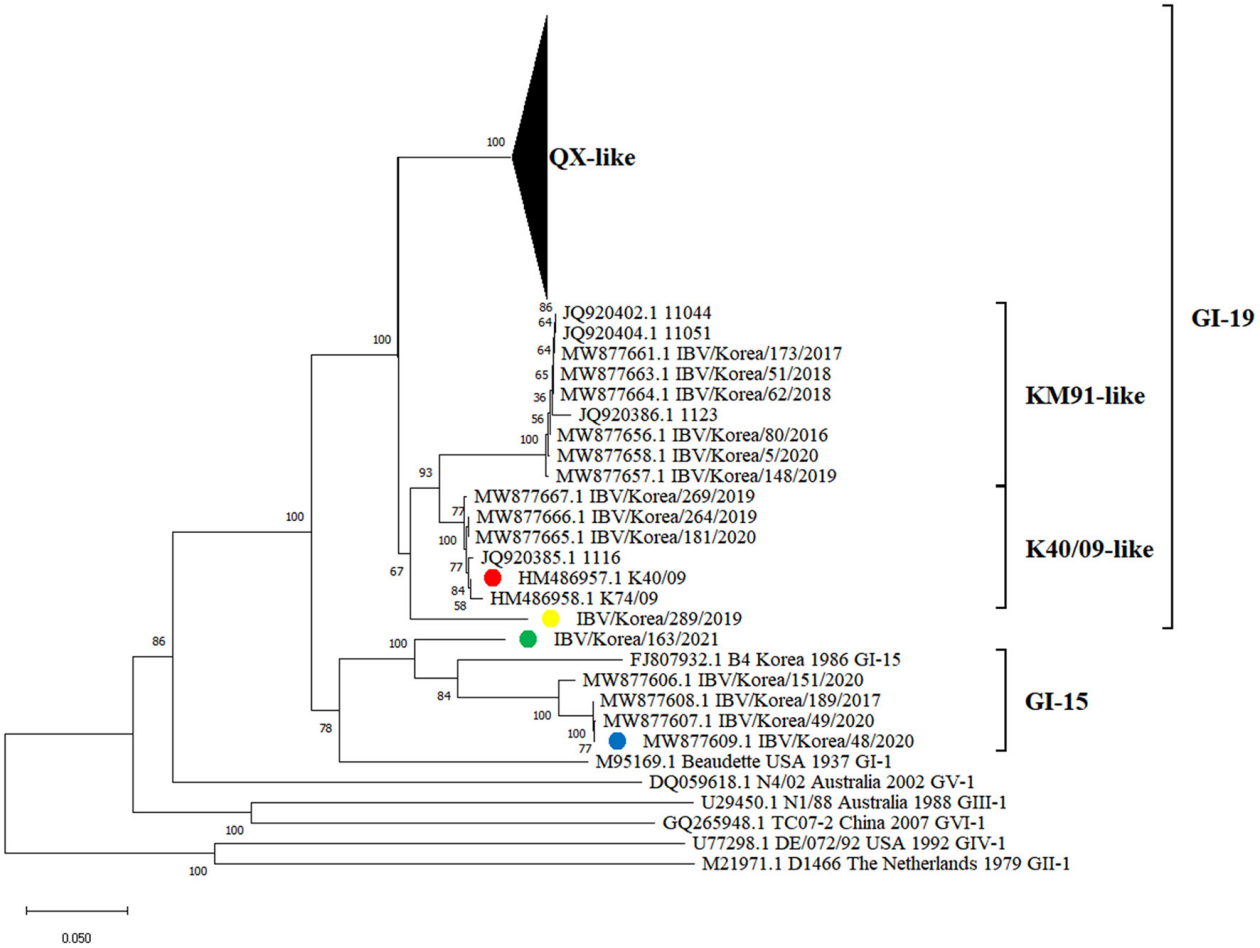
Phylogenetic tree based on the S1 gene nucleotide sequences of IBVs. The IBVs newly isolated in this study are highlighted in yellow and green circles (plural). Strains highlighted in red and blue circles (plural) are putative parental strains of isolates, which are identified as progenitors of recombination events using RDP4 software in this study.

Phylogenetic analysis of the S1 gene revealed that the K289/19 and K163/21 viruses were genetically distinct from previously identified IBVs in South Korea. These viruses did not belong to the QX-like, KM91-like, or K40/09-like and GI-15 subgroups in the phylogeny (Figure 1).

**Figure 2:**
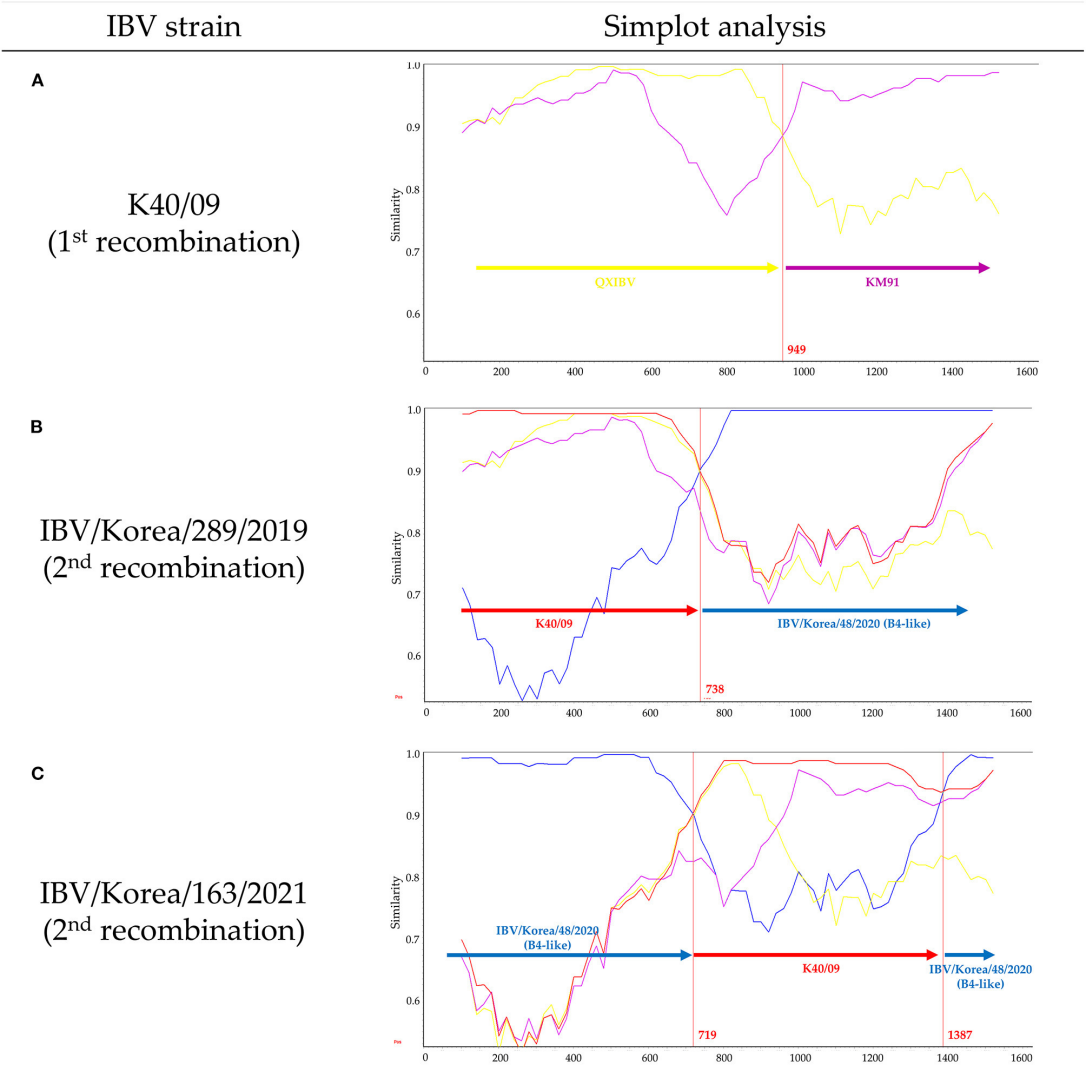
Evolutionary history of novel IBVs produced by multiple recombination events in Korea. Simplot analysis was performed with a window size of 200 bp and a step size of 20 bp. **(A)** Similarity plot of K40/09 strain, which was reported as recombinant between QXIBV (yellow) and KM91 (purple). **(B)** Similarity plot of K289/19. **(C)** Similarity plot of K163/21. Putative parental strains are highlighted in red (K40/09 strain) and blue (IBV/Korea/48/2020 strain). Red vertical lines and numbers indicate the crossover breakpoints and their nucleotide positions.

Recombination events in K289/19 and K163/21 were identified using RDP4 software (Table 1). Five different methods in RDP4 and Simplot analysis showed that their putative parental strains are most likely the K40/09 strain of GI-19 and the IBV/Korea/48/2020 strain of GI-15 (p value < 1 × 10^−14^) (Figure 2). Other putative recombination events were excluded, because they did not satisfy the criteria for recombination event described above (*p* < 1 × 10^−14^ at least five detection methods).

**Table 1:** Recombinant detection of S1 gene in IBV isolated in this study.

**Recombinant strain**	**Major parental strain**	**Minor parental strain**	***p*** **-values of the detection methods** ^**a**^				

			**RDP**	**GENECONV**	**MaxChi**	**Chimera**	**3Seq**
IBV/Korea/289/2019	K40/09 (99.6%)	IBV/Korea/48/2020 (100%)	5.56 × 10^−49^	3.97 × 10^−39^	4.71 × 10^−30^	4.68 × 10^−30^	4.46 × 10^−81^
IBV/Korea/163/2021	IBV/Korea/48/2020 (99.0%)	K40/09 (98.9%)	7.43 × 10^−44^	2.29 × 10^−37^	9.56 × 10^−28^	1.04 × 10^−27^	1.79 × 10^−71^

^a^Recombination events were confirmed when P-values were < 1 × 10^−14^ from at least five detection methods. The major and minor parental strain is the virus contributing the larger fraction of the recombinant sequences and the smaller fraction of the recombinant sequences to the generation of recombinant IBV, respectively.

The K40/09 strain is a recombinant strain derived from QXIBV and KM91 (21) (Figure 2A). Single crossover breakpoint in the S1 gene of K289/19 virus was identified at the nucleotide position 738 bp; 1–738 bp was similar to the K40/09 strain, followed by IBV/Korea/48/2020 strain (Figure 2B). However, two crossover breakpoints were identified in the S1 gene of K163/21 virus, at nucleotide positions 719 bp and 1387 bp; the 719–1387 bp region in the S1 gene of IBV/Korea/48/2020 strain was substituted by the partial S1 gene of K40/09 strain (Figure 2C). Briefly, the S1 genes of two isolates were produced by recombination between same parental strains, the K40/09 strain and IBV/Korea/48/2020, but in different patterns.

**Figure 3:**
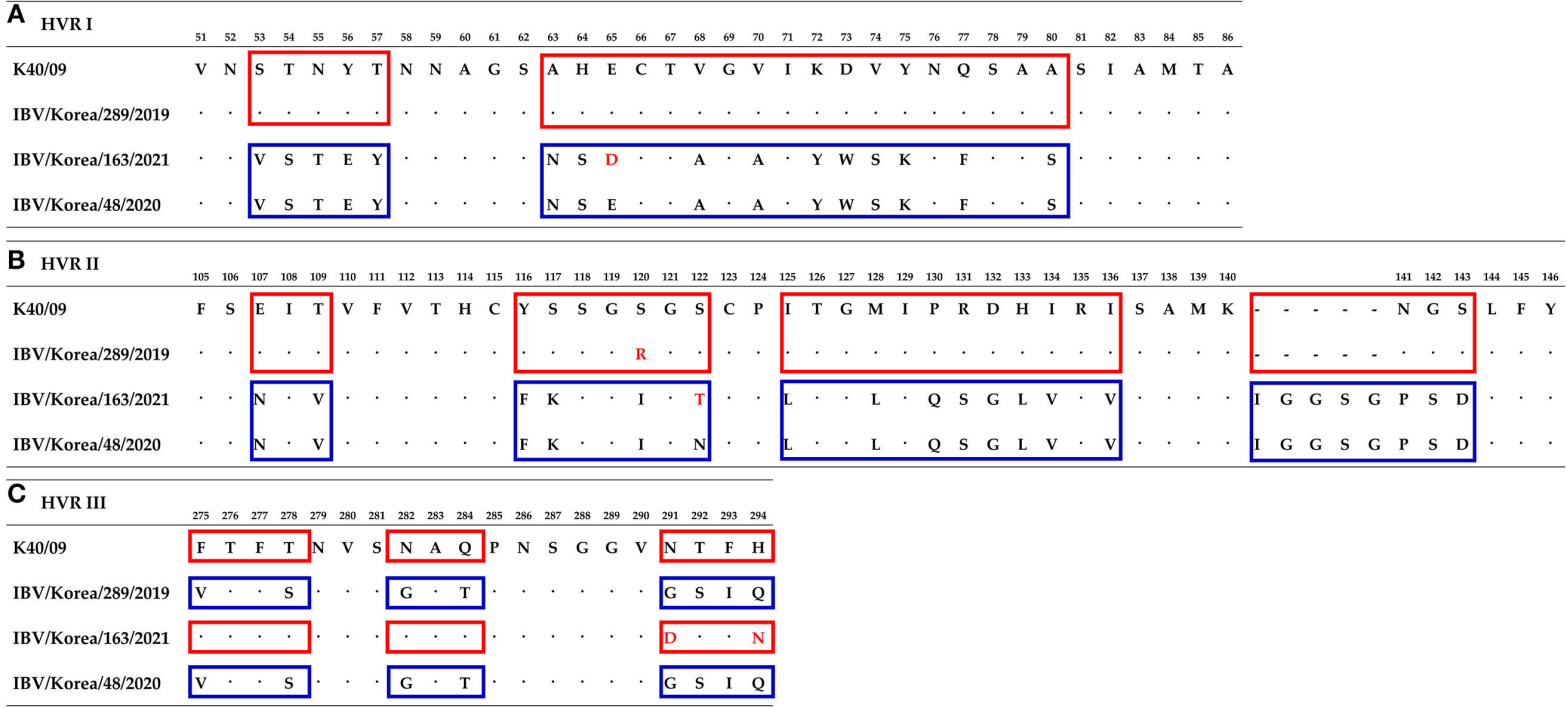
Comparison of amino acids sequences of hypervariable region at S1 gene between isolates and their parental strains. Numbering is based on isolate K289/19. **(A)** Hypervariable region I. **(B)** Hypervariable region II. **(C)** Hypervariable region III. Similar amino acids sequences with the K40/09 strain are highlighted in red boxes. Blue boxes indicate amino acids sequences similar to the IBV/Korea/48/2020 strain. Substitutions of amino acids are highlighted with red font.

The nucleotide sequence identity of the S1 gene of each recombinant was compared with the GI-15 and GI-19 IBVs identified previously in Korea. Nucleotide sequence identities of K289/19 were 81.9–87.8% with the GI-15 IBVs, and 85.9–91.6% with the GI-19 IBVs. Nucleotide sequence identities of K163/21 were 84.6–91.6% with the GI-15 IBVs, and the 78.7–86.8% with GI-19 IBVs (data not shown). Sequence analysis of the HVR I, II, and III in the S1 gene showed that the HVR I and II of K289/19 were similar to the K40/09 strain, except one substitution at 120th amino acid (S120R), but HVR III was similar to the IBV/Korea/48/2020 strain (Figure 3). In contrast, the HVR I and II of the isolate K163/21 was similar to the IBV/Korea/48/2020 strain, except two substitutions at 65th and 122th amino acids (E65D and N122T). The HVR III of the isolate K163/21 was similar to the K40/09 strain except two substitutions at 291th and 294th amino acids (N291D and H294N).

### 3.2. Pathogenicity investigation and tissue tropism

**Figure 4:**
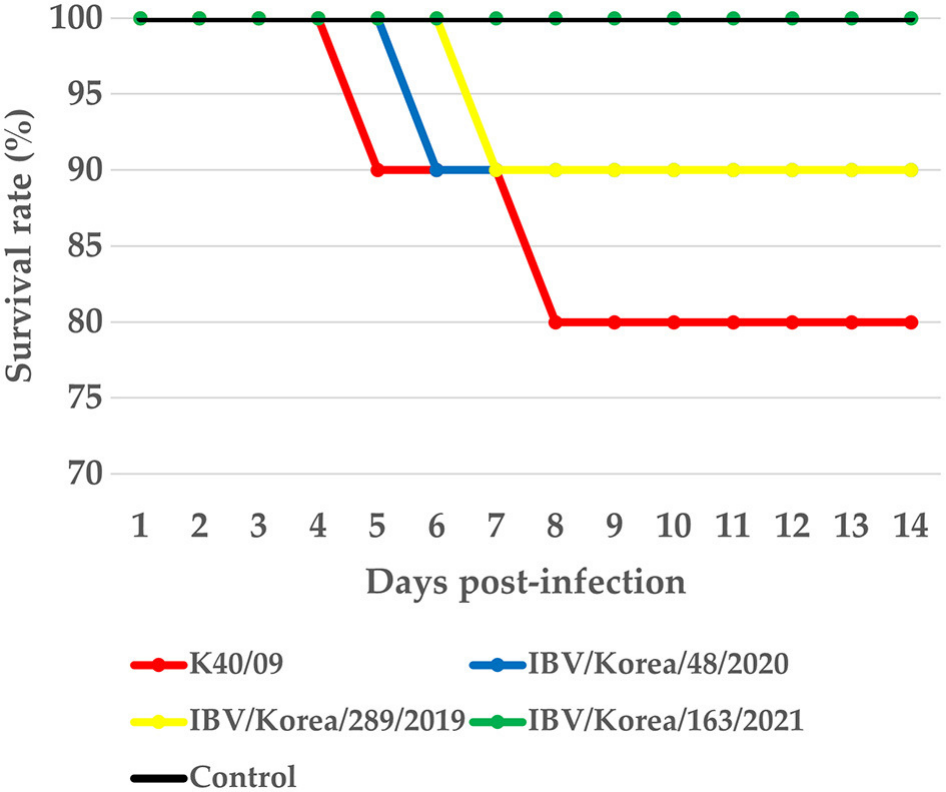
Survival rate of chickens infected with IBV isolates.

Mortality was not observed in the K163/21 challenged group or the negative control group (Figure 4). The chickens challenged with the IBV/Korea/48/2020 or K289/19 viruses exhibited 10% mortality (1/10) at 6 dpi and 7 dpi, respectively. The chickens inoculated with K40/09 strain showed 20% mortality (2/10). Mild exudates and petechial hemorrhage in the trachea were commonly observed in the dead chickens. Nephritis and urate deposition were only observed in the dead chickens challenged with the K289/19 and the K40/09 viruses, indicating nephropathogenicity of these viruses.

**Figure 5:**
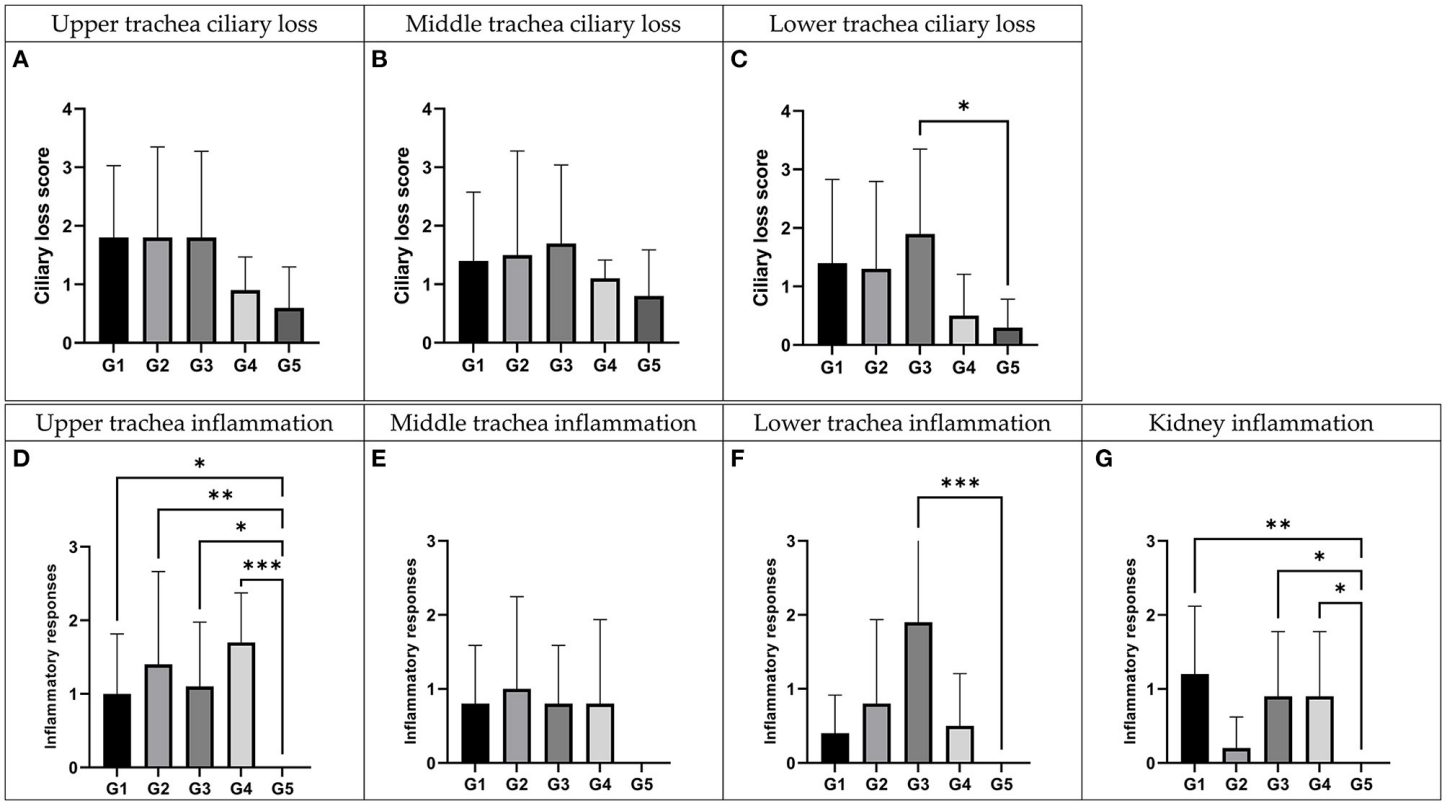
Histopathologic examination of recombinant strains and their parent strains. Trachea and kidney samples were collected at 5 days post-inoculation. G1: K40/09 challenged group, G2: IBV/Korea/48/2020 challenged group, G3: IBV/Korea/289/2019 challenged group, G4: IBV/Korea/163/2021 challenged and G5: negative control group. **(A–C)** Ciliary damages were compared with negative control. **(D–G)** Inflammation responses were estimated comparing with negative control. ^*^P < 0.05, ^**^P < 0.01, and ^***^P < 0.001, compared to the negative control group as determined using Dunnett's multiple comparisons test.

Histopathological examination (Figure 5) and virus re-isolation test (Table 2) were performed at 5 dpi to evaluate the pathogenicity and tissue tropism of each virus. For the ciliary loss score of upper trachea, the score of the K40/09, IBV/Korea/48/2020, K289/19 group were higher than the negative control group, followed by the score of the K163/21 group. However, there was no statistical difference compared to the negative control group (*p* > 0.05). Likewise, for the ciliary loss score of middle trachea, the scores of inoculated groups were higher than that of the negative control group, but there was no significant difference compared with negative control group. However, for the ciliary loss score of lower trachea, the score of K289/19 challenged group was the highest (p<0.05), followed by the K40/09 challenged group and the IBV/Korea/48/2020 challenged group. The K163/21 had a slightly higher score compared to the negative control group. Based on the results of the scoring of ciliary loss, it seems that the K289/19 was more pathogenic than its parental strains, but the K163/21 was less pathogenic than its parental strains.

**Table 2:** Virus re-isolation rate in the trachea and kidney.

**Inoculation strain**	**No. of inoculation virus isolated/no. of inoculated**	

	**Trachea**	**Kidney**
K40/09	10/10^****^	10/10^****^
IBV/Korea/48/2020	10/10^****^	0/10
IBV/Korea/289/2019	10/10^****^	9/10^****^
IBV/Korea/163/2021	10/10^****^	9/10^****^
PBS(Negative control)	0/10	0/10

^****^P < 0.0001 compared to the negative control group as determined using one-tailed Fisher's exact test.

All the groups inoculated with each virus showed significantly higher inflammation score than the negative control group in the upper trachea (*P* < 0.05). Chickens inoculated with each virus showed higher inflammation score than the negative control group in the middle trachea, but there was no statistically significant difference (*P* > 0.05). For the inflammation score in the lower trachea, the score of K289/19 challenged group was only significantly higher than the negative control group (*P* < 0.001). Collectively, all inoculated viruses were identified to be pathogenic in trachea, although virulence of each virus was slightly different. The inflammation score in kidney tissues of IBV/Korea/48/2020 challenged group was similar to the negative control group. However, the K40/09 group and two novel recombinant viruses showed statistically higher inflammation scores in kidney compared to the negative control group (*P* < 0.05).

Similar results were also identified from the virus re-isolation test (Table 2). The positive rate of IBV was 100% in trachea of all groups, suggesting efficient infection and robust replication of all IBVs in the upper respiratory tract of chickens. However, the positive rates for the kidney tissues varied among the groups. Chickens inoculated with K40/09 showed a 100% re-isolation rate from kidney tissue samples. Chickens infected with K289/19 or K163/21 exhibited 90% positive rate from kidney tissues. However, the IBV/Korea/48/2020 group and the negative control group showed 0% positive rate from kidney tissues.

### 3.3. Antigenicity study using cross-neutralization test

Cross-neutralization tests were performed using the recombinant viruses and their putative parental strains. NI values retrieved from the cross-neutralization tests were converted to R-values using the calculation method described by Archetti and Horsfall (35). R-values implicate antigenic relatedness between two viruses. R-values >70% indicate the same serotype, R-values between 33 and 70% indicate subtype with a minor difference, between 11 and 32% indicate a subtype with a major difference, and R-values <11% indicate a different serotype (19). Recombinant viruses showed different antigenic properties as shown in Table 3. The two parental strains, K40/09 and IBV/Korea/48/2020, were antigenically different (7%). The isolate K289/19 was antigenically close with the K40/09 strain (76%), but different with the IBV/Korea/48/2020 strain (10%). The antigenic identity of K163/21 was identified to be major subtype difference from K40/09 strain (25%) and minor subtype difference from K289/19 (36%) and IBV/Korea/48/2020 (42%).

**Table 3:** Antigenic relatedness (R-value) between the IBVs used in this study.

**Virus**	**Antisera**			
	**K40/09 (GI-19 K40/09-like)**	**IBV/Korea/48/2020** **(GI-15 B4-like)**	**IBV/Korea/289/2020 (recombinant)**	**IBV/Korea/163/2021** **(recombinant)**
K40/09 (GI-19 K40/09-like)	100^a^	-	-	-
IBV/Korea/48/2020 (GI-15 B4-like)	7	100	-	-
IBV/Korea/289/2019 (recombinant)	76	10	100	-
IBV/Korea/163/2021 (recombinant)	25	42	36	100

^a^R > 70%: little or no difference in antigenic identity; 33–70%: minor subtype difference; 11–32%: major subtype difference; 0–10%: different serotypes.

## 4. Discussion

The IBV evolves through a variety of mutation mechanisms, not only accumulations of point mutations but also recombination events, which makes it difficult to perfectly prevent the infection of IBVs (3–5). In particular, the recombination between field strains and vaccine strains contributes to the sudden emergence of various strains (36). In this study, we isolated two novel recombinant IBVs (K289/19 and K163/21) and investigated the genetic characteristics based on the S1 gene and pathobiological features in chickens. Our data showed that these viruses were produced by recombination between same progenitors, the GI-19-like virus and GI-15-like virus in different patterns. Since two recombinants were identified as progenies of identical parental strains based on the S1 gene, we had assumed that these viruses may have similar pathobiological characteristics to one of their parental strains. However, the pathogenicity, tropism, and antigenicity were not identical to those of parental strains. Although the S1 subunit is a major determinant of pathogenicity, tissue tropism, and antigenicity of IBV (8–12), genetic changes in S1 gene could not explain molecular mechanism of these phenotypical changes since other genes such as S2 gene and replicase gene can also alter their pathobiological characteristics (37, 38). Whole genome sequencing would be required to better understand the molecular mechanism for these findings.

The HVRs of S1 gene are highly diverse and often associated with antigenicity, pathogenicity, and tissue tropism (13, 39). In particular, it has been reported that the HVR II is associated with nephropathogenicity (14). The K289/19, harboring HVR II derived from the nephropathogenic IBV, exhibited kidney affinity. Unexpectedly, the K163/21, harboring HVR II derived from the non-nephropathogenic IBV, also showed nephropathgenicity in chickens. It implies that the HVR II of S1 gene is not the only factor that determine the tissue tropism of IBV. Furthermore, other studies have suggested that genetic changes in other structural or non-structural protein genes could contribute to the pathogenicity and tissue tropism of coronaviruses (14, 37, 40–42). Therefore, further studies are needed to clarify the determinants of pathogenicity and tissue tropism.

Live attenuated vaccines derived from GI-15 and GI-19-like viruses have been used in Korea (30, 43). The antigenicity of the recombinant viruses was identified to be different, despite of being derived from the same parental strains. Since IBVs generally show poor cross-protective efficacy between different serotypes (44), vaccine strains should be carefully selected based on the epidemiology and antigenic properties of IBVs. It should be also noted that the pathogenicity and antigenicity of the recombinant virus in field condition could be different from laboratory animal study and insufficient efficacy of vaccines could accelerate viral evolution (45). Recently, an IBV that has highly similar recombinant pattern with the K163/21 virus was reported in Korea, which has the partial S1 gene of QX-like IBV(GI-19) between 724 and 1,102 bp in the S1 gene of GI-15 IBV (29). The emergence of such a diverse range of natural recombinant IBVs raises a concern that novel recombinants could possibly escape vaccine induced immunity and extensively spread in Korea possibly due to insufficient cross-neutralization between commercial vaccines and novel variants. Therefore, enhanced surveillance for newly emerged recombinants should be carried out, and IBV characteristics such as whole genome sequences, pathogenicity, and antigenicity should be quickly analyzed to prepare control measures.

## Data availability statement

The original contributions presented in the study are included in the article/Supplementary material, further inquiries can be directed to the corresponding authors.

## Ethics statement

The animal study was reviewed and approved by Konkuk University IACUC.

## Author contributions

C-SS: conceptualization, validation, and project administration. D-HL, H-JK, H-CL, and AC: methodology. H-JK, Y-JC, and HL: formal analysis. D-HL, H-JK, Y-JC, and HL: investigation. H-JK and H-CL: resources. H-JK and AC: data curation. H-JK: writing—original draft preparation and visualization. D-HL, H-CL, AC, and C-SS: writing—review and editing. D-HL and C-SS: supervision and funding acquisition. All authors have read and agreed to the published version of the manuscript.
