# Supplementary material for: Novel recombinant avian infectious bronchitis viruses from chickens in Korea, 2019–2021
Source: Front Vet Sci. 2023 Feb 1;10:1107059. doi: 10.3389/fvets.2023.1107059 (PMC9931148; doi:10.3389/fvets.2023.1107059)
Supplement: Supplementary file 1 [file Table_1.DOCX]

**Supplementary Table 1.** Novel recombinant IBVs and reference strains used in this study

| **Virus** | **Nation** | **Year** | **Genotype** | **Accession number** |
| --- | --- | --- | --- | --- |
| IBV/Korea/289/2019 | South Korea | 2019 | Recombinant strain | OP886678 |
| IBV/Korea/163/2021 | South Korea | 2021 | Recombinant strain | OP886679 |
| K40/09 | South Korea | 2009 | GI-l9 | HM486957 |
| IBV/Korea/48/2020 | South Korea | 2020 | GI-15 | MW877609 |
| DE/072/92 | USA | 1992 | GIV-1 | U77298 |
| D1466 | The Netherlands | 1979 | GII-1 | M21971 |
| N1/88 | Australia | 1988 | GIII-1 | U29450 |
| TC07-2 | China | 2007 | GVI-1 | GQ265948 |
| N4/02 | Australia | 2002 | GV-1 | DQ059618 |
| K154/05 | South Korea | 2005 | GI-19 | FJ807922 |
| QIA-KR/D79/05 | South Korea | 2005 | GI-19 | KU900740 |
| K463/04 | South Korea | 2004 | GI-19 | FJ807924 |
| K283/04 | South Korea | 2004 | GI-19 | FJ807923 |
| FJ807931 | South Korea | 2004 | GI-19 | FJ807931 |
| K1255/03 | South Korea | 2003 | GI-19 | FJ807928 |
| K1019/03 | South Korea | 2003 | GI-19 | FJ807927 |
| IBV/Korea/63/2020 | South Korea | 2020 | GI-19 | MW877613 |
| IBV/Korea/96/2020 | South Korea | 2020 | GI-19 | MW877618 |
| IBV/Korea/95/2020 | South Korea | 2020 | GI-19 | MW877615 |
| IBV/Korea/111/2020 | South Korea | 2020 | GI-19 | MW877616 |
| IBV/Korea/224/2019 | South Korea | 2019 | GI-19 | MW877612 |
| IBV/Korea/46/2020 | South Korea | 2020 | GI-19 | MW877622 |
| IBV/Korea/135/2019 | South Korea | 2019 | GI-19 | MW877619 |
| IBV/Korea/24/2018 | South Korea | 2018 | GI-19 | MW877640 |
| IBV/Korea/183/2018 | South Korea | 2018 | GI-19 | MW877626 |
| IBV/Korea/29/2018 | South Korea | 2018 | GI-19 | MW877627 |
| IBV/Korea/61/2018 | South Korea | 2018 | GI-19 | MW877631 |
| IBV/Korea/25/2018 | South Korea | 2018 | GI-19 | MW877630 |
| IBV/Korea/22/2018 | South Korea | 2018 | GI-19 | MW877629 |
| IBV/Korea/18/2018 | South Korea | 2018 | GI-19 | MW877628 |
| IBV/Korea/59/2016 | South Korea | 2016 | GI-19 | MW877642 |
| IBV/Korea/17/2018 | South Korea | 2018 | GI-19 | MW877644 |
| IBV/Korea/064/2018 | South Korea | 2018 | GI-19 | MW877643 |
| 11035 | South Korea | 2011 | GI-19 | JQ920398 |
| 11026 | South Korea | 2011 | GI-19 | JQ920396 |
| 11045 | South Korea | 2011 | GI-19 | JQ920403 |
| 11038 | South Korea | 2011 | GI-19 | JQ920400 |
| 11039 | South Korea | 2011 | GI-19 | JQ920401 |
| 11036 | South Korea | 2011 | GI-19 | JQ920399 |
| IBV/Korea/166/2016 | South Korea | 2016 | GI-19 | MW877654 |
| IBV/Korea/87/2016 | South Korea | 2016 | GI-19 | MW877651 |
| K74/09 | South Korea | 2009 | GI-19 | HM486958 |
| IBV/Korea/269/2019 | South Korea | 2019 | GI-19 | MW877667 |
| 1116 | South Korea | 2010 | GI-19 | JQ920385 |
| IBV/Korea/264/2019 | South Korea | 2019 | GI-19 | MW877666 |
| IBV/Korea/181/2020 | South Korea | 2020 | GI-19 | MW877665 |
| 1123 | South Korea | 2010 | GI-19 | JQ920386 |
| IBV/Korea/80/2016 | South Korea | 2016 | GI-19 | MW877656 |
| IBV/Korea/5/2020 | South Korea | 2020 | GI-19 | MW877658 |
| IBV/Korea/148/2019 | South Korea | 2019 | GI-19 | MW877657 |
| IBV/Korea/62/2018 | South Korea | 2018 | GI-19 | MW877664 |
| IBV/Korea/51/2018 | South Korea | 2018 | GI-19 | MW877663 |
| 11051 | South Korea | 2011 | GI-19 | JQ920404 |
| 11044 | South Korea | 2011 | GI-19 | JQ920402 |
| IBV/Korea/173/2017 | South Korea | 2017 | GI-19 | MW877661 |
| B4 | South Korea | 1986 | GI-15 | FJ807932 |
| IBV/Korea/151/2020 | South Korea | 2020 | GI-15 | MW877606 |
| IBV/Korea/189/2017 | South Korea | 2017 | GI-15 | MW877608 |
| IBV/Korea/49/2020 | South Korea | 2020 | GI-15 | MW877607 |
| Beaudette | USA | 1937 | GI-1 | M95169 |
